# A novel xylene-free deparaffinization method for the extraction of proteins from human derived formalin-fixed paraffin embedded (FFPE) archival tissue blocks

**DOI:** 10.1016/j.mex.2014.07.006

**Published:** 2014-08-08

**Authors:** Anthony Mansour, Rajaa Chatila, Noha Bejjani, Carole Dagher, Wissam H. Faour

**Affiliations:** a School of Medicine, Lebanese American University, Byblos, Lebanon; b Lebanese American University Medical Center – Rizk Hospital, Beirut, Lebanon

**Keywords:** Deparaffinization of paraffin embedded tissue blocks without the use of xylene, Pathology, Paraffin blocks, Protein extraction, Western blot, Xylene, Water

## Abstract

Protein detection methods in formalin-fixed paraffin embedded (FFPE) tissue blocks are widely used in research and clinical setting in order to diagnose or to confirm a diagnosis of various types of diseases. Therefore, multiple protein extraction methods from FFPE tissue sections have been developed in this regard. However, the yield and the quality of proteins extracted from FFPE tissues are significantly reduced in blocks stored for longer periods of time. Regardless the protein extraction method used, tissue sections must be first deparaffinized with xylene, and then washed in serial dilutions of ethanol in order to remove the toxic organic solvent “xylene” and rehydrate the tissue. The objective of this study was first to develop a method to deparaffinize FFPE blocks that excludes the use of toxic solvent “xylene”. Second minimize the time required to perform the extraction. Here we describe a method where:

• The entire paraffin embedded blocks are deparaffinized and rehydrated using only hot distilled water as a substitute for both xylene and ethanol
• The entire procedure takes about 15 min
• Deparaffinized blocks are immediately homogenized in lysis buffer, and the obtained lysate analyzed by Western blot.

With this new modified technique, we were able to successfully detect actin and AKT proteins in lysates from blocks embedded in paraffin for up to 9 years.

## Graphical abstract

**Figure:**
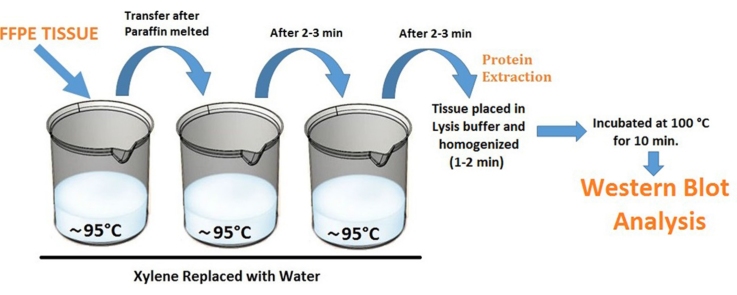

## Method details

The protein extraction procedure from FFPE blocks includes the following steps: (a) Samples collection; (b) Deparaffinization of the FFPE tissue blocks with hot distilled water; (c) Tissue homogenization in homogenization buffer; (e) Western blot analysis.

**(a)** **Samples collection**
  We used a total of 22 human colorectal cancer tissue blocks processed (formalin fixation in 10% neutral buffered formalin) in the pathology laboratory at the University Medical Center-Rizk Hospital (UMC-RH) from years 2006–2008 (colorectal cancer) and March of 2014 (breast cancer). The research project was approved by the Institutional Review Board (IRB) at the Lebanese American University.
**(b)** **Deparaffinization of FFPE tissue blocks**
  The deparaffinization process was achieved with hot distilled water [Paraffin wax melt at temperature around 70 °C [1] replacing all steps that include xylene and serial ethanol washes]. The below procedure is optimized to deparaffinize a small section or the entire paraffin-embedded tissue blocks and is performed as follow:
  (1) Place three clean beakers containing distilled water on a temperature controlled hot plate and heat until temperature is almost 90–95 °C.
  (2) Once the above temperature is reached, place FFPE tissue block in the first beaker until the paraffin block melts completely and the tissue becomes visible.
  (3) Transfer the tissue using clean forceps to the second beaker containing hot distilled water (90–95 °C) and keep for additional 2–3 min, and then finally transfer the tissue to the third beaker and incubate for another 2–3 min. These successive steps will ensure that all paraffin have melted off the tissue sample.
**(c)** **Homogenization of the deparaffinized block in lysis buffer**
  **(i)** **Protocol**
    (1) Prepare in advance fresh homogenization buffer as follow: mix 40 μl of 100 mM PMSF prepared previously with 2 ml of the lysis buffer
    (2) Place 100 mg of the tissue on a clean slide glass on ice and gently cut the sample into small pieces of about 1 mm^3^ size
    (3) Transfer the pieces of tissue into a clean tube and add 250 μl of the freshly prepared homogenization buffer
    (4) Homogenize the sample using a homogenizer (or mini-homogenizer if the tissue sample is small, a sonicator–homogenizer can be used as well) until the pieces are fully homogenized and a homogenous solution is obtained.
    (5) Transfer the homogenate into clean eppendorf and heat at 100 °C for 10 min.
    (6) Add an equal volume of 2× Laemmli sample buffer to the sample.
    (7) Add 3% (v/v) of β-mercaptoethanol to the sample
    (8) Incubate the samples at 100 °C for 10 min.
    (9) Centrifuge the sample at a speed of 4000 rpm for 2 min at 4 °C. Collect the supernatant containing the proteins and transfer into new clean eppendorf.
    (10) Measure total protein concentration of each sample
    (11) Add 0.2% bromophenol blue to the sample
    (12) Load 25 μl of the sample on acrylamide gel for mini Western blot analysis and compared to control protein isolated from cultured MDA231 cell lines, or stored at −20 °C for later use
  **(ii)** **Buffers used in the experiment are prepared as follow:**
    (1) 10 ml of lysis buffer containing the following components
      • 50 mM Tris–HCl adjusted to a pH = 7.4 (0.5 ml of 1 M)
      • 1% Triton-X (10 μl of stock)
      • 0.2% Sodium deoxycolate (50 mg)
      • 1 mM disodium EDTA (20 μl 0.5 M)
      • 0.2% SDS (100 μl of SDS 20%)
      • Adjust the final volume with H_2_O to obtain 10 ml
    (2) **PMSF buffer**
      • Prepare 100 mM of PMSF (phenylmethylsulfonyl fluoride) by adding 174 mg in 10 ml of ethanol, isopropanol or methanol and directly store at −20 °C
    (3) **10** **ml of 2× Laemmli sample buffer are prepared by mixing the following components:**
      • 1.2 ml of 1 M Tris–HCl (pH = 6.8)
      • 4 ml of glycerol (50%)
      • 4 ml of 10% SDS
      • 0.8 ml H_2_O

*Note*: All remaining amounts of buffers can be stored at −20 °C.

### Western blot

Western blot was performed to validate the success of the protein extraction from the FFPE tissues. Protein extracts were separated on SDS denaturing 10% polyacrylamide gel and electrophoretically transferred to polyvinylidene difluoride membranes (PVDF). The membranes were blocked with 5% skim milk for 1 h, washed, and then probed with primary antibodies raised against actin and Akt-1 (Abcam) proteins at 4 °C overnight. Finally the membranes were treated with HRP-coupled secondary antibodies for 1 h, and the membranes were then washed twice with TBST (1×) for 30 min each thereafter. Protein detection is revealed using the ECL detection kit (Abcam plc, 330 Cambridge Science Park, Cambridge, UK). Blot images were obtained with the Image Lab Software (BioRad, Chemidoc imaging instrument).

### Figure preparation

**Fig. 1:**
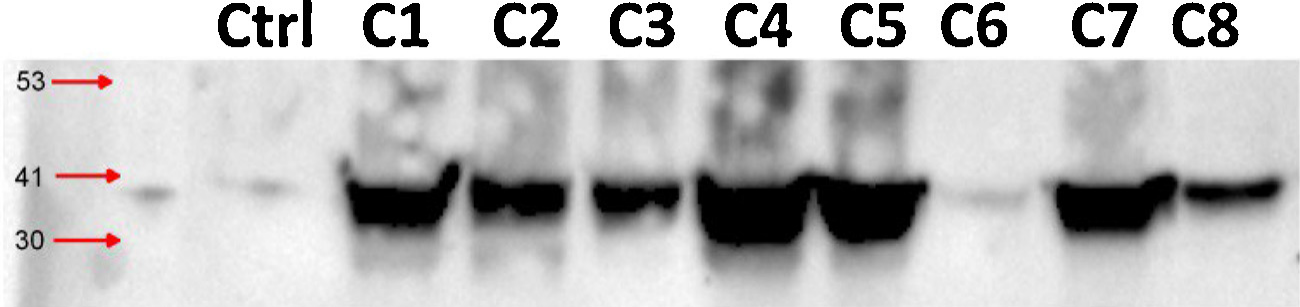
Protein expression of actin in different colorectal cancer FFPE tissues. Eight colorectal cancer FFPE blocks labeled C1–C8 were deparaffinized, homogenized and protein are extracted according to the protocol described above, a total of 25 μl of each sample is resolved using Western blotting analysis which demonstrated the expression of the protein Actin using anti-actin antibody and compared to control actin isolated from MDA 231 cell lines.

**Fig. 2:**
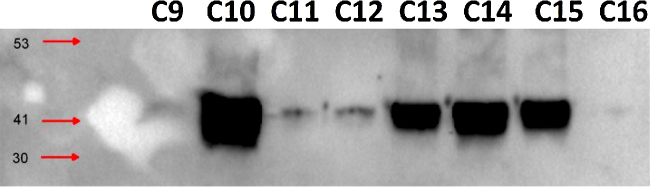
Eight colorectal cancer FFPE blocks labeled C9–C16 were deparaffinized, homogenized and protein are extracted according to the protocol described above, a total of 25 μl of each sample is resolved using Western blotting analysis which demonstrated the expression of the protein Actin.

**Fig. 3:**
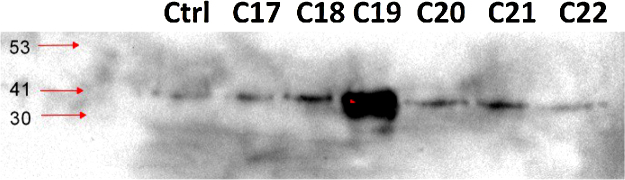
Six colorectal cancer FFPE blocks labeled C17–C22 were deparaffinized, homogenized and protein are extracted according to the protocol described above, a total of 25 μl of each sample is resolved using Western blotting analysis which demonstrated the expression of the protein Actin compared to control actin isolated from MDA 231 cell lines. Molecular weight (in kDa) is shown on the left side of the figure.

**Fig. 4:**
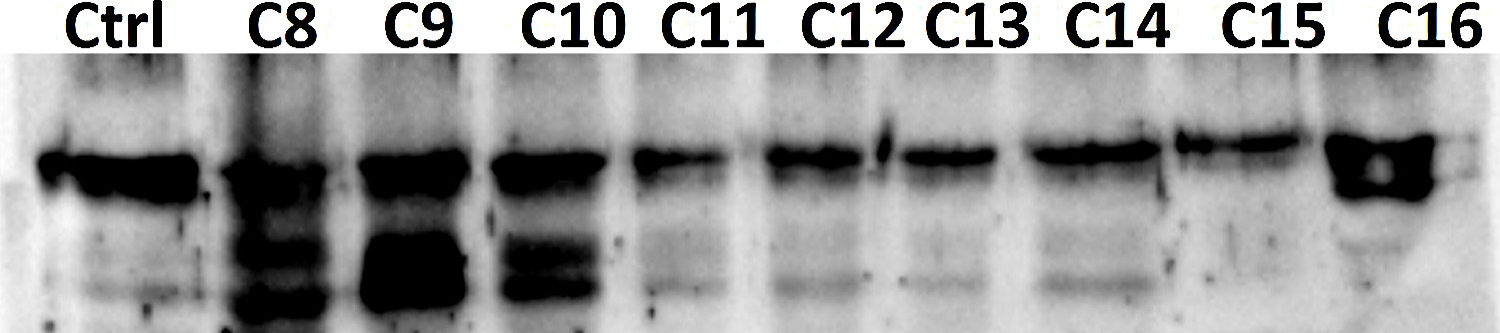
Nine colorectal cancer FFPE blocks labeled C8–C16 were deparaffinized, homogenized and protein are extracted according to the protocol described above, a total of 25 μl of each sample is resolved using Western blotting analysis which demonstrated the expression of the protein AKT using anti-AKT antibody and compared to control AKT isolated from MDA 231 cell lines.

Figures are prepared with Photoshop and cropped images are shown for each corresponding figure (Figs. 1–4).

## Additional information

### Background

FFPE tissues are the most abundant human resources available in the labs worldwide, accounting from more than one billion samples, and are considered to be of great value in retrospective molecular studies. These tissues are routinely used for the diagnosis of a variety of diseases. Moreover, they play a very important role in medical research due to the availability of the clinical and histopathological data of every patient. The use of formaldehyde to preserve the morphology of tissues created a barrier for many analytical procedures since formaldehyde crosslinks proteins, DNA and RNA. Xylene, a flammable and toxic organic solvent, is the only available solvent used to deparaffinize tissues prior to staining with Hematoxylin or eosin (H&E) staining. Xylene inhalation can affect the central nervous system causing headaches, nausea, vomiting and dizziness [2]. Isolation of intact non-degraded protein from FFPE is of considerable importance which renders available clinical diagnostic tools and immense amount of clinical data. However, the fixation process is largely associated with protein degradation and cross-linking, making conventional protein analysis ineffective. Most of the developed methods focused on using protein extract for proteimic evaluation which requires a “clean intact soup of protein extract”. All previous studies depended on reversing the steps in the preparation of the FFPE tissues for the deparaffinization of the tissue blocks and extraction of DNA, RNA and proteins [3,4]. In addition, these methods are based upon the use of xylene as deparaffinization solvent, and takes longer time for the extraction to be achieved with a yield that depend upon the age of the FFPE and the storage conditions [5,6]. For instance Becker et al. has described an optimal solution for the extraction and analysis of proteins from FFPE tissues known as the commercial Qproteome Kit [3]. Consistent with improving protein isolation methods several methods for protein extraction from FFPE tissues have been developed for proteomic analysis and elegantly summarized in an expert review describing various studies that replaced xylene with a variety of organic or ionic solvents [7,8]. The solvent used included heptane, octane, toluene and mineral as replacement to xylene. However, these studies were characterized by various rate of success in term of yield of protein isolation [8]. Moreover, several factors may influenced optimal yield and protein quality isolated from FFPE. These factors include pH, temperature, lysis buffer. Studies showed that pH range of 8–9 yielded the best results. However, Fowler et al. showed that more than 80% of total protein extraction is achieved at pH 4 [9]. Consequently, sodium dodecyl sulfate (SDS) was the prefer lysis buffer used to dissolve deparaffinized specimen, the concentration used ranged from 0.1 to 2%. Finally, temperature can play an important role in increasing the yield of extraction since heating might break intramolecular and intermolecular covalent cross-links and partial thermal hydrolysis of methylene bridges [10]. Using this method, pathologist can benefit of working within a biofriendly environment and avoid exposure to hazardous solvent.

### Further observations

Isolation of intact protein from FFPE is a major challenge to the clinical community. While xylene is a hazardous solvent to be used in labs, it represents the only available choice for deparaffinization a key limiting step before protein extraction procedure. However the use of xylene is associated with reduced protein amount and integrity. Although multiple successful attempts to replace xylene exist in the literature [7,8], these newly developed methods have not been fully standardized to satisfy guidelines and reproducibility for clinical use. At first attempt in our study we succeeded to develop a protein extraction method using biofriendly solvent other than xylene. Then we optimized the methods in order to be used in human samples, and finally we performed the test on archival tissues embedded in paraffin for at least 9 years. Several considerations were taken into account during the development of the method including avoiding working with toxic solvent, integrity and yield of isolated protein and most importantly the reproducibility of the results [11]. Moreover, other protein extraction parameters can influence efficient protein isolation such as ischemic time (>60 min), fixation time with formal and storage duration [8,12]. In our study, we were unable to determine the duration of ischemic or the fixation time but we can assume to be relatively minimal and similar to all specimens since collected specimens are directly transferred to the clinical pathology lab soon after surgery. This latter assumption is considered adequate since these specimens are used to clinically diagnose cancer. Storage time of paraffin embedded specimen can largely vary from tissue to another. The samples we tested were stored for more than 9 years before tissue processing. Alternatively, there no approved consensus to describe duration at which paraffin blocks are considered “long-term or archival”. Several studies considered storing specimen in paraffin for few days as archival blocks [13]. Interestingly, it was shown that storage of paraffin blocks for up to 10 years did not affect protein profiling. As such, it seems to be that dehydration and paraffin embedding has no significant negative effect on protein recovery compared to the above described parameters [14]. Therefore, by replacing xylene with water avoid multiple steps necessitating the use of multiple organic and non-organic solvents. Reducing the exposure of the deparaffinized blocks will certainly improve the yield and the quality of the extracted proteins. In order to deparaffinize the blocks we exposed the specimen to nearly boiling water which will lead to fast dissolution of paraffin. High temperature might help breaking down bond and crosslinks formed during the preservation procedure. Even though the specimens were exposed to high temperature but it was for a very short duration (around 2–3 min) enough to ensure complete dissolution of paraffin without damaging the liberated specimen. Short exposure can damage a thin superficial outer layer of the entire blocks while the deep tissues remain considerably intact and protected from heat. This particularity was evident where results obtained with large block provided similar results compared to small specimens. It remains to be determined the optimal duration and temperature at which a block should be incubated. The amount of proteins isolated using our techniques varied from 14 μg protein/mg tissue which can be considered acceptable and similar to that documented in the literature [8,13]
